# Molecular spectrum of *TP53* mutations in plasma cell dyscrasias by next generation sequencing: an Italian cohort study and overview of the literature

**DOI:** 10.18632/oncotarget.7241

**Published:** 2016-02-08

**Authors:** Marta Lionetti, Marzia Barbieri, Martina Manzoni, Sonia Fabris, Cecilia Bandini, Katia Todoerti, Filomena Nozza, Davide Rossi, Pellegrino Musto, Luca Baldini, Antonino Neri

**Affiliations:** ^1^ Department of Oncology and Hemato-Oncology, University of Milan, Milan, Italy; ^2^ Hematology Unit, Fondazione IRCCS Ca' Granda, Ospedale Maggiore Policlinico, Milan, Italy; ^3^ Laboratory of Pre-Clinical and Translational Research, IRCCS-CROB, Referral Cancer Center of Basilicata, Rionero in Vulture (PZ), Italy; ^4^ Department of Translational Medicine, Division of Hematology, Amedeo Avogadro University of Eastern Piedmont, Novara, Italy

**Keywords:** multiple myeloma, plasma cell leukemia, TP53, next-generation sequencing, mutational analysis

## Abstract

The prevalence of *TP53* mutations greatly varies between tumor types; in multiple myeloma (MM) they were rarely detected at presentation, while increased frequency was reported with disease progression. Using next-generation sequencing, we analyzed *TP53* exons 4-9 in a large representative cohort comprising patients with MM at diagnosis and more aggressive forms of plasma cell (PC) dyscrasia, identifying mutations in 4/129 (3%) MM, 6/24 (25%) primary PC leukemia, and 2/10 (20%) secondary PC leukemia cases. A similar increase in prevalence associated with disease aggressiveness (5%, 29.2% and 44%, respectively) was observed for *TP53* deletion. Interestingly, in five patients mutations were not concomitant with *TP53* deletion. Furthermore, longitudinal analysis revealed the acquisition of *TP53* mutations in three of nineteen cases analyzed at relapse. Identified variants were mostly missense mutations concentrated in the DNA binding domain, only partly reflecting the pattern globally observed in human cancers. Our data confirm that *TP53* mutations are rare in MM at presentation and rather represent a marker of progression, similarly to del(17p); however, their occurrence even in absence of deletions supports the importance of their assessment in patients with PC dyscrasia, in terms of both risk stratification and therapeutic implications.

## INTRODUCTION

The tumor suppressor protein p53, encoded by the *TP53* gene at chromosome 17p13, mediates the response to various stress signals (including DNA damage, oxidative stress, ribonucleotide depletion, and deregulated oncogene expression), many of which are encountered during tumor development and malignant progression [1]. Loss of p53 function, due to *TP53* deletions and/or mutations or by defects in the signalling pathways upstream or downstream of p53, is associated with oncogenesis, cancer progression and drug resistance. *TP53* is mutated in about half of human cancers, and the prevalence of gene mutations greatly varies between different tumor types.

Recently, whole exome sequencing (WES) analyses in multiple myeloma (MM) [2–5], albeit reporting slightly higher mutational frequencies (probably for the extension of the analysis to the entire coding sequence and the greater sensitivity), confirmed the findings of the early studies [6–10], i.e. that *TP53* mutations are relatively rare at presentation (mutation prevalence ranging from 0% to 9.7% in representative MM patients' cohorts). The frequency of mutations increases with disease stage, reaching 25-30% in plasma cell leukemia (PCL) [11, 12], and 80% in human myeloma cell lines (HMCLs) [13].

A strong association has been described between *TP53* mutation and del(17p) [14]. Deletions, predominantly monoallelic, of chromosome 17p13 region containing the *TP53* gene locus occur in about 10% of untreated MM cases [15–17]; the incidence rate reported in PCL ranges from 35% to 75% [12, 18], and is particularly high (more than 50%) in HMCLs [19]. 17p13 deletion confers a very negative effect on survival [20], displaying the most powerful cutoff for predicting survival if the deletion is carried by more than 50% of malignant plasma cells [21]. Finally, a recent study identified *TP53* as the critical gene of 17p13 deletion in MM [22].

## RESULTS

**Figure 1:**
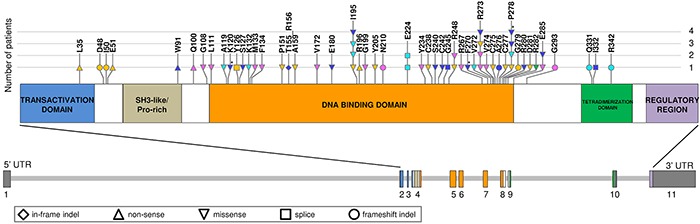
Representation of *TP53* mutations identified in MM/PCL patients, including overview of the literature Amino acid changes caused by mutations are indicated on the scheme of the structural organization of TP53 protein. In the lower part of the figure, schematizing *TP53* gene, exons are numbered under the boxes, filled with the color corresponding to the encoded domain. Somatic mutations identified in the present series (blue) and in the main recent MM datasets are depicted (Chapman *et al*, 2011, and Lohr *et al*, 2014, yellow; Walker *et al*, 2012, green and Walker *et al*, 2015, pink; Bolli *et al*, 2014, light blue). Mutations identified in the present cohort in patients at relapse are marked by an asterisk. Symbols of mutations are shaped according to their type (see legend in the figure).

We performed next generation sequencing (NGS) of *TP53* exons 4-9 on genomic DNA of 151 primary patients with plasma cell dyscrasia, including 129 MM and 12 primary PCL (pPCL) patients at diagnosis, and 10 secondary PCL (sPCL) cases (median depth of coverage = 162x). The mutational analysis was limited to this portion of the gene coding sequence based on the fact that it contains almost 98% of *TP53* mutations identified in the main published whole genome and exome sequencing studies in MM [2–5]. Twelve additional pPCL samples have been recently subjected to WES analysis [11]. Globally, in the 163 tested patients we identified 14 non-synonymous somatic variants in 12 cases (Table 1, Figure 1). Ten mutations were single nucleotide variations (SNVs), all of which but one (introducing a premature stop codon in PCL-037) were missense mutations. The remaining four mutations were nucleotide deletions involving 2, 6, 10 and 82 base pairs respectively, only one of which (6-bp deletion in PCL-037) caused an amino acid deletion without alteration of the reading frame. Exon 8 was the most frequently targeted by mutations (5 variants), followed by exons 5 and 7 (3 variants each), and exons 4, 6 and 10 (one variant each), whereas no variants were found in exon 9. Apart from the nonsense mutation W91* in case PCL-037 (localized in the SH3-like/Pro-rich structural motif) and the 10-nt deletion spanning intron 9-exon 10 junction in case PCL-017, all other variants targeted the DNA binding domain. In regards to the four nucleotide deletions identified, only one (T155_R156del in PCL-037) is reported in the IARC p53 website (http://www-p53.iarc.fr/index.html) [23], and none has been described in other MM series. In contrast, all ten single nucleotide variations found by us are listed in the dataset of somatic mutations in sporadic cancers of the IARC database. Each of the variants is carried by a single patient in our cohort.

**Table 1:** Summary of *TP53* non-synonymous/indel variants identified by NGS in the present dataset

Variant^°^	cDNA position (NM_000546.5)	Amino acid change	IARC mut ID^‡^	Domain_function^‡^	Structural _motif^‡^	Transactivation class^‡^	Mutated samples (VAF)
17:7673803G > A	c.817C > T	R273C	3730	DNA binding	L1/S/H2	non-functional	PCL-030 (100%)
17:7674947A > G	c.584T > C	I195T	2446	DNA binding	NDBL/beta-sheets	non-functional	PCL-027 (90.9%)
17:7673787G > A	c.833C > T	P278L	3817	DNA binding	L1/S/H2	non-functional	PCL-018 (96.1%)
17:7670714 _7670723del	c.994-8 _995del	altered splicing	/	/	/	/	PCL-017 (70%)
*17:7673810A > T*	*c.810T > A*	*F270L*	*3682*	*DNA binding*	*NDBL/beta-sheets*	*non-functional*	*MM-281_2 (96.8%)*
17:7674245T > C	c.718A > G	S240G	3146	DNA binding	L2/L3	non-functional	MM-262 (100%)
17:7673781C > T	c.839G > A	R280K	3849	DNA binding	L1/S/H2	non-functional	PCL-031 (98.52%)
17:7675074C > T	c.538G > A	E180K	2229	DNA binding	L2/L3	partially functional	PCL-012 (95.82%)
17:7675232G > T	c.380C > A	S127Y	1339	DNA binding	L1/S/H2	non-functional	MM-375 (93.33%)
17:7673767C > T	c.853G > A	E285K	3932	DNA binding	L1/S/H2	non-functional	PCL-004 (72.71%)
17:7675144 _7675149delGCGGGT	c.463 _468delACCCGC	T155_R156del	1785	DNA binding	NDBL/beta-sheets	NA	PCL-037 (49.25%)
17:7676096C > T	c.273G > A	W91*	922	SH3-like/Pro-rich	SH3-like/Pro-rich	NA	PCL-037 (47.34%)
*17:7673788G > T*	*c.832C > A*	*P278T*	*3810*	*DNA binding*	*L1/S/H2*	*non-functional*	*MM-280_2 (26.28%)*
*17:7676011T > C*	*c.358A > G*	*K120E*	*1245*	*DNA binding*	*L1/S/H2*	*non-functional*	*PCL-038 (22.11%)*
17:7674149 _7674230del	c.733 _782+32del	altered splicing	/	/	/	/	MM-213 (8.22%)
17:7674238C > T	c.725G > A	C242Y	3185	DNA binding	L2/L3	non-functional	MM-343 (10.96%)
17:7673794 _7673795delCA	c.825 _826delTG	A276Lfs*29	/	/	/	/	MM-213 (2.65%)

° Genomic positions based on hg38.

‡ Data were obtained from the IARC p53 website (http://www-p53.iarc.fr/index.html). Variants indicated in italic were detected in the sample collected at the second timepoint in longitudinally analyzed patients. VAF: variant allele frequency

Taking advantage of the most recent WES reports in MM [2–5], we globally considered the molecular spectrum of *TP53* mutations including the results of our present series. We evidenced that mutations cluster in the DNA binding domain; I195 and R273 are the most frequently mutated residues (I195T detected in three cases and I195M in one; R273H in two patients, and R273L and R273C each in one case), followed by P278 (P278S recurring twice, and P278L once), R248 (R248Q and R248W in one patient each) and E285 (E285K in two cases). Contrary to what is globally observed in MM, somatic mutations at codon 195 are not among the most recurrent in human tumors. Notably, the substitution I195T, like other tumorigenic mutations in the β-sandwich affecting hydrophobic residues in the protein core, was reported to be severely destabilizing [24], determining a half-life of the unfolding of the mutant protein of less than one minute at 37°C [25]. Conversely, R273 is one of the six residues identified as hotspots for tumorigenic mutations in human cancers; it is generally targeted by two predominant substitutions, i.e. R273H and R273C, both of which identified in MM cases and preventing DNA binding by the isolated core domain [24]. With regard to the amino acid residue 278, it is one of the essential amino acids for major groove contacts in the pentamer sequence of the consensus DNA binding site [26]. The substitution E285K is the most common *TP53* temperature-sensitive mutation (its wild type TP53 activity is reconstituted at about 32°C) and is also carried by the RPMI-8226 MM cell line [13, 27, 28]. Amino acid substitutions R→W and R→Q at the hot spot codon 248 both inactivate the transactivating functions of wild type TP53, although it has been demonstrated that they differently contribute to *in vitro* malignant behavior of tumor cells through gain-of-function properties [29]. Other codons are targeted by single mutational events in each patient's dataset. Furthermore, virtually all the missense mutations identified in our study are classified as non-functional [23] in the IARC database (see Materials and Methods).

Noteworthy, we detected the occurrence of two concomitant *TP53* mutations in two patients of our dataset. In particular, PCL-037 carried the nonsense mutation W91* at a variant allele frequency (VAF) of 47.3% and a 2-aa deletion (T155_R156del) at a VAF of 49.2%, suggesting the occurrence of both mutations virtually in the entire tumor population (due to the experimental design, it is not possible to determine whether the mutations are carried on two different chromosomes or on the same allele); and MM-213 harbored a 82 nt-deletion (at VAF of 8.2%) comprising the last 50 nucleotides of exon 7 and the first 32 nucleotides of intron 7 and putatively originating an in frame amino acid deletion and altered splicing (due to the loss of a splice donor site), and a two-nucleotide deletion (at VAF of 2.6%) in exon 8 causing frameshift (A276Lfs*29).

**Figure 2:**
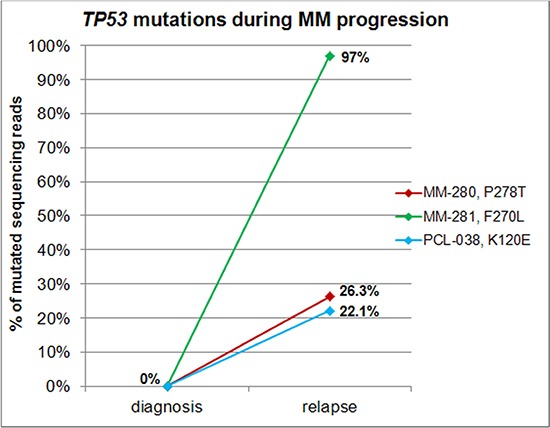
*TP53* mutations during disease progression Variants reported in the legend were acquired during disease progression, being detectable at plotted allele frequencies only in the late sample of longitudinally analyzed patients.

Mutation frequencies were of 3.1% (4/129), 25% (6/24), and 20% (2/10) in MM, pPCL and sPCL patients, respectively (Freeman-Halton test, *P* = 0.0005), in line with the notion that *TP53* mutations are associated with aggressive forms of plasma cell dyscrasias and have a role in tumor progression than initiation [6, 7]. This hypothesis was further supported by *TP53* NGS analysis of 19 of our patients at relapse/leukemic transformation, revealing the acquisition of non-functional missense mutations (undetectable in the corresponding samples analyzed by NGS at diagnosis) in the DNA binding domain in three cases (Table 1, Figures 1 and 2): specifically, the longitudinal analysis detected the substitutions P278T (VAF = 26.3%) in the myeloma patient MM-280 at relapse, F270L (VAF = 97%) at the time of leukemic transformation in the myeloma patient MM-281, and K120E (VAF = 22.1%) in the pPCL patient PCL-038 at relapse, respectively. Notably, genome-wide DNA copy number data were also available for MM-281, indicating two interstitial deletions of 17p arm not involving *TP53* locus present at sub-clonal level in the sample at onset, and then detecting a larger deletion spanning from 17p12 to 17pter in virtually all cells of the later sample (Supplementary Figure S1).

**Figure 3:**
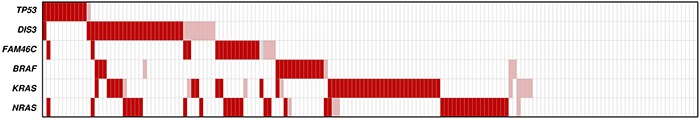
Heat map distribution of *TP53*, *DIS3*, *FAM46C*, *BRAF*, *KRAS* and *NRAS* gene mutations among MM/PCL patients The rows correspond to the indicated genes, and columns represent individual MM or PCL samples, which are color-coded on the basis of gene status (white: wild-type; light red: Sanger-undetectable mutations; dark red: Sanger-detectable mutations).

We also tested the association between the occurrence of *TP53* mutations and common genetic abnormalities in MM (i.e. chromosomal translocations at *IGH* locus, hyperdiploidy, deletions at chromosome regions 13q, 17p, and 1p, and gain at 1q), for which the entire patients' cohort was characterized by means of fluorescence *in situ* hybridization (FISH) (Tables 2 and 3). *IGH* translocations (particularly *MAF*-translocations) were found to be more frequent in *TP53*-mutated cases compared with the whole series (92% *versus* 50%, *P* = 0.0024). *TP53* mutations were negatively associated with hyperdiploid status (no hyperdiploid cases among the *TP53*-mutated ones *versus* 33% in the entire cohort, *P* = 0.009); the over-representation of PCL patients within mutated cases could affect these findings, although in part confirmed in other series [30]. However, the low *TP53* mutation prevalence in MM did not allow to limit contingency analyses to this class. As regards 17p deletion, a strong association of gene mutation with the deletion of the remaining allele was observed: in fact, deletion of *TP53* locus globally affected 10.6% of cases [4.7% (6/128) of MM, 29.2% (7/24) of pPCL and 44.4% (4/9) of sPCL patients respectively], while its prevalence reached 58% in *TP53*-mutated subset (*P* < 0.0001). Overall, *TP53* inactivation as a result of gene deletion and/or mutation occurred in 7% of MM, 37.5% of pPCL and 44.4% of sPCL patients; in particular, the two lesions were simultaneously present respectively in 0.8%, 16.7% and 22.2% of cases, causing biallelic gene inactivation. A fraction of *TP53* mutations co-occurred with variants affecting other MM driver genes (Figure 3) [31–33]. Indeed, two *TP53*-mutated cases carried also mutations in *DIS3*, and another one was simultaneously mutated in *TP53*, *NRAS* and *FAM46C* genes. However, *TP53* mutations were not positively associated with those of any other gene; on the contrary, the absence of *TP53* mutations in *KRAS*-mutated cases was deemed statistically significant (*P* = 0.019).

**Table 2:** Clinical and molecular characteristics of the 12 MM/PCL patients carrying *TP53* mutations

*Sample*	*Disease stage^§^*	Variant^$^	Mutated reads (%)	AA change	*t(4; 14)*	*t(11; 14)*	*t(14; 16)*	*t(14; 20)*	del (13)^*^	del (17p)^*^	1q gain^*^	1p loss^*^	*HD*^‡^
MM-213	MM	17:7674149 _7674230del (82)	8.2%	altered splicing	-	+	-	-	+	-	-	-	-
		17:7673794 _7673795del CA	2.6%	A276L fs*29									
MM-262	MM	17:7674245T>C	100%	S240G	-	-	-	-	-	-	+	-	-
MM-343	MM	17:7674238C>T	11%	C242Y	-	+	-	-	-	-	-	-	-
MM-375	MM	17:7675232G>T	93.3%	S127Y	+	-	-	-	-	+	-	-	-
PCL-004	pPCL	17:7673767C>T	72.7%	E285K	-	-	+	-	-	-	-	-	-
PCL-012	sPCL	17:7675074C>T	95.8%	E180K	-	-	+	-	-	+	-	-	-
PCL-017	pPCL	17:7670714 _7670723del	70%	altered splicing	-	-	+	-	+	+	+	+	-
PCL-018	pPCL	17:7673787G>A	96.1%	P278L	-	+	-	-	+	+	-	-	-
PCL-027	pPCL	17:7674947A>G	90.9%	I195T	-	+	-	-	-	+	-	-	-
PCL-030	pPCL	17:7673803G>A	100%	R273C	-	-	-	+	+	+	-	-	-
PCL-031	sPCL	17:7673781C>T	98.5%	R280K									
PCL-037	pPCL	17:7675144_7675149delGCGGGT	49.2%	T155_R156del	-	-	+	-	-	+	+	-	-
		17:7676096C>T	47.3%	W91^*^									

§ MM: multiple myeloma; pPCL: primary plasma cell leukemia; sPCL: secondary plasma cell leukemia.

$ Genomic positions based on hg38.

* del(13), del(17), 1p loss and 1q gain were determined by FISH.

‡ HD: presence of the hyperdiploid status on the basis of FISH evaluation criteria.

**Table 3:** Clinical and molecular characteristics of the 163 MM/PCL patients analyzed for *TP53* mutations

Characteristic	All patients (n = 163)		*TP53*-wild type (n = 151)		*TP53*-mutated (n = 12)		*P* value^a^
	n	%	n	%	n	%	
MM	129	*79*	125	*83*	4	*33*	0.0005
pPCL	24	*15*	18	*12*	6	*50*	
sPCL	10	*6*	8	*5*	2	*17*	
del(13q)	76	*47*	71	*47*	5	*42*	n.s.
chr 13 disomic patients	86	*53*	79	*53*	7	*58*	
del(17p)	17	*11*	10	*7*	7	*58*	<0.0001
17p disomic patients	144	*89*	139	*93*	5	*42*	
1q gain	65	*42*	62	*44*	3	*25*	n.s
1q disomic patients	89	*58*	80	*56*	9	*75*	
1p loss	18	*13*	17	*13*	1	*8*	n.s.
1p disomic patients	123	*87*	112	*87*	11	*92*	
*IGH* translocation	80	*50*	69	*46*	11	*92*	0.0024
no *IGH* translocation	81	*50*	80	*54*	1	*8*	
hyperdiploid	49	*33*	49	*36*	0	*0*	0.009
non-hyperdiploid	100	*67*	88	*64*	12	*100*	

a Significance was assessed by Freeman-Halton extension of Fisher's exact test for disease type, and by Fisher's exact test for all other variables. *n.s.*: not significant.

**Figure 4:**
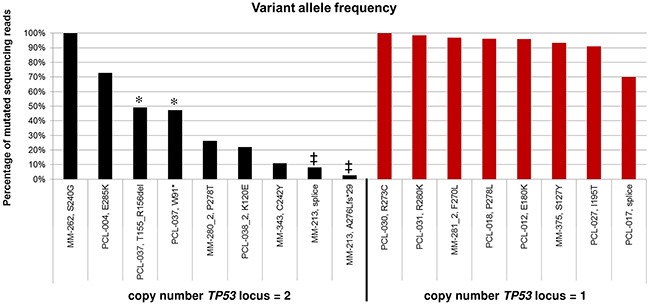
Allele frequency of the *TP53* mutations identified by NGS analysis Horizontal axis: sample id and carried amino acid variant are reported; patients are ordered according to copy number of *TP53* locus (as determined by FISH analysis) and decreasing VAF. In case of multiple mutations carried by an individual patient, the bars of the histogram are marked by the same symbol.

Concerning variant allele frequencies, in mutated patients positive for del(17p) VAFs were almost always indicative of the exclusive presence of the mutated allele; on the contrary, in mutated cases at onset and disomic for chromosome 17 VAFs ranged from 2.65% to 100%, suggesting the occurrence of minor mutated subclones in MM-213 and MM-343; the presence of clonal heterozygous mutations in PCL-037; the predominance of mutated allele in PCL-004; and apparent copy number-neutral loss of heterozigosity in MM-262 (Figure 4). As stated above, in the three patients longitudinally analyzed and showing in the late sample the emergence of *TP53* mutations not visible in the earlier sample, VAFs were of 22.1%, 26.3% and 97%, respectively.

## DISCUSSION

In the present study, the use of deep NGS in a large representative cohort of MM at diagnosis and more aggressive disease stages substantially confirmed the accepted notion that *TP53* mutations in MM at presentation are rarely detected, while their frequency increases with disease progression. The enhanced sensitivity of our analysis, in fact, was not accompanied by a substantial increase in the mutation rate observed in MM cases, consistent with the fact that we did not find many mutations at particularly low allele frequency, which could be undetectable by the traditionally used Sanger method. Although we cannot exclude that an even greater depth of coverage would eventually bring out further smaller mutated subclones, this finding is in line with what reported by other recent NGS studies [2, 3, 34]. In extra-medullary MM, *TP53* inactivation was significantly more frequent, as a result of both gene mutations and deletions, confirming that these lesions represent late-progression events. Consistently, we noticed the emergence of a mutation in three of 19 MM/PCL patients analyzed at relapse; despite the NGS analysis of the earlier samples, we could not detect at onset minor mutant subclones potentially responsible for relapse. Concerning deletion of chromosome arm 17p, its frequency in intra-medullary MM patients of our series appears to be lower than that generally reported, a finding that could be related to different sample size and patient selection criteria; a strong, albeit not absolute, association of gene mutation with the deletion of the remaining allele was observed also in our series, in line with previously reported data [30]. As regards pPCL, it is noteworthy that a recent WES analysis by our group indicated that *TP53* impairment seemed to lie within a more generalized alteration of cell cycle checkpoints, which involved other genes among which the most frequently mutated were *ATM*, *ATR*, *CDKN1A* and *BRCA1* [11]. Furthermore, although less prevalent in MM, mutations in *ATM* or *ATR* genes were lately reported to be associated with a negative impact on survival of MM patients enrolled onto the NCRI Myeloma XI Trial, in which the inability to deliver an effective apoptotic response to DNA damage represented the most significantly prognostic mutational marker [5].

Concerning the type of variants, unlike Chng *et al*. [30] and more similarly to what reported by Lodé and colleagues [14] and other more recent papers [2, 3, 5], most of the mutations identified here were single nucleotide missense mutations. Differently from many tumor suppressor genes, in human cancers *TP53* is actually most commonly altered by missense mutations, that not only lead to a loss/diminution of the wild type TP53 activity, but, since TP53 normally acts as a tetramer, may also function as dominant negative inhibitors over any remaining wild type TP53 activity, or even give rise to a more aggressive tumor profile through gain of function activity. Gain of function roles in different cellular processes have indeed been demonstrated for several TP53 mutants also detected in MM/PCL of the present and other published datasets [2–5] (P151S, R248Q, R248W, R273H, R273C, V274F, P278S, and R280K) [35]. MM-associated *TP53* mutations are concentrated in the DNA binding domain, and the frequency at which amino acid residues are targeted only in part reflects the one globally observed in human cancers.

In conclusion, our data provide further evidence that mutations of *TP53* are rare in MM at diagnosis and rather represent a marker of progression, as well as the deletion of the locus. This would be consistent with the hypothesis that defective TP53 may cause extramedullary disease [20]. Surely, alterations of *TP53* identify a high-risk population of patients, even in the context of molecularly comprehensive risk scoring models [5]. Although the close association between mutations and deletions makes it difficult to understand the prognostic contribution of individual lesions [2, 14, 30], the recently demonstrated haploinsufficiency of the gene [22] may be responsible of TP53 pathway deficiency whenever the cells don't have two intact copies of the gene (as a result of both deletion and mutations). Furthermore, taking into consideration the dominant negative effect or gain of function of many of *TP53* mutations, TP53 functionality appears to be even more compromised. In general, the occurrence of mutations even in cases not carrying the deletion supports the importance of their assessment in MM patients, given that many anti-MM drugs induce apoptosis through multiple pathways that are at least in part dependent upon functional TP53 activation.

## MATERIALS AND METHODS

### Patients

The study cohort consisted of a retrospective series of 129 multiple myeloma (MM) and 8 primary plasma cell leukemia (pPCL) cases at onset, and 10 secondary PCL (sPCL), admitted to our Institution from July 2001 to April 2014. Additional 16 pPCL patients belonged to a prospective multicenter clinical trial (RV-PCL-PI-350, EudraCT N°2008-003246-28) [36]. All the patients included in this study have been previously described [33].

### Sample preparation and molecular analyses

Highly purified (≥90% in all cases) plasma cells (PCs) were obtained from bone marrow specimens (collected at the time of diagnosis) using CD138 immunomagnetic microbeads as previously described [37, 38]; 19 cases were re-sampled at relapse/leukemic transformation. Genomic DNA was extracted using Wizard genomic purification DNA kit (Promega Corporation).

### Mutation analysis

NGS of *TP53* exons 4-9 (RefSeq NM_000546.5, representing the longer transcript encoding the longest protein isoform) was performed on genomic DNA using the Genome Sequencer Junior instrument (Roche-454 Life Sciences, Penzberg, Germany), as previously described [39]. Further details on primer sequences and sequencing protocol are available in the Supplementary Materials and Methods and Supplementary Table S1. The obtained sequencing reads were mapped to the *TP53* human reference sequence (RefSeq NC_000017.11) and analyzed by the Amplicon Variant Analyzer (AVA) software version 3.0 (454 Life Sciences) to establish the variant allele frequency.

The presence of each obtained non-synonymous variant was verified in an independent PCR product by conventional sequencing whenever the sensitivity of the Sanger method was consistent with the VAF. NGS analysis was repeated in case of mutations detected in less than 10% of sequencing reads. To exclude germline variants, we sequenced the matched normal DNA, when available, or consulted the International Agency for Research on Cancer (IARC) Database (http://www-p53.iarc.fr). Information about the functional relevance of the detected mutations was obtained from IARC database; specifically, a TP53 mutant was classified as “non-functional” if the median of its transcriptional activities measured in reporter assays on eight different promoters containing TP53-binding sequences was lower than 20% compared to the wild type protein.

### Statistical analysis

Contingency analyses were made using two-sided Fisher's exact test (*P* value lower than 0.05 was considered significant).

## SUPPLEMENTARY FIGURE AND TABLE


