# Supplementary material for: Molecular spectrum of TP53 mutations in plasma cell dyscrasias by next generation sequencing: an Italian cohort study and overview of the literature
Source: Oncotarget. 2016 Feb 8;7(16):21353–61. doi: 10.18632/oncotarget.7241 (PMC5008290; doi:10.18632/oncotarget.7241)
Supplement: Supplementary file 1 [file oncotarget-07-21353-s001.pdf]

SUPPLEMENTARY FIGURE AND TABLE

Mutation analysis

Genomic DNA was extracted using Wizard genomic purification DNA kit (Promega Corporation, Madison, WI, USA) according to manufacturer's instructions, spectrophotometrically quantified and amplified using FastStart High Fidelity Polymerase (Roche) and fusion primers (Roche) containing M13 adapter sequences and the sequence-specific primers (Supplementary Table 1) spanning *TP53* exons 4–9 (RefSeq NM_000546.5, representing the longer transcript encoding the longest protein isoform).

Amplicon library A and B sequencing adapters and multiplex identifier (MID) tags were then added to both tails of amplicons by a second amplification step. PCR conditions were as follows: in the first amplification step, denaturation step at 94°C for 5 min followed by 25 cycles at 94°C (30 sec. per cycle), annealing step at 58°C (30 sec. per cycle), and extension at 72°C (45 sec. per cycle), followed by a final extension at 72°C for 7 min.; in the second amplification step, denaturation step at 94°C for 5 min followed by 25 cycles at 94°C (20 sec. per cycle), annealing step at 55°C (20 sec. per cycle), and extension at 72°C (45 sec. per cycle), followed by a final extension at 72°C for 10 min.. PCR products were visualized on agarose gel, purified using AMPure XP DNA-binding paramagnetic beads (Agencourt Bioscience Corp., Beckman Coulter S.p.A, Milan, Italy), and quantified using picogreen dye (Life Technologies, Carlsbad, California) and the Victor X2 (Perkin Elmer, Waltham, Massachusetts) fluorometer. Samples were then pooled together at equimolar ratios to prepare for Roche/454 pyrosequencing. The obtained amplicon library was added to the emulsion PCR at a ratio of 0.8 molecules per bead and subjected to deep sequencing on the Genome Sequencer Junior instrument (Roche-454 Life Sciences). The obtained sequencing reads were mapped to the *TP53* human reference sequence (RefSeq NC_000017.11) and analyzed by the Amplicon Variant Analyzer (AVA) software version 3.0 (Roche-454 Life Sciences) to establish the mutant allele frequency.

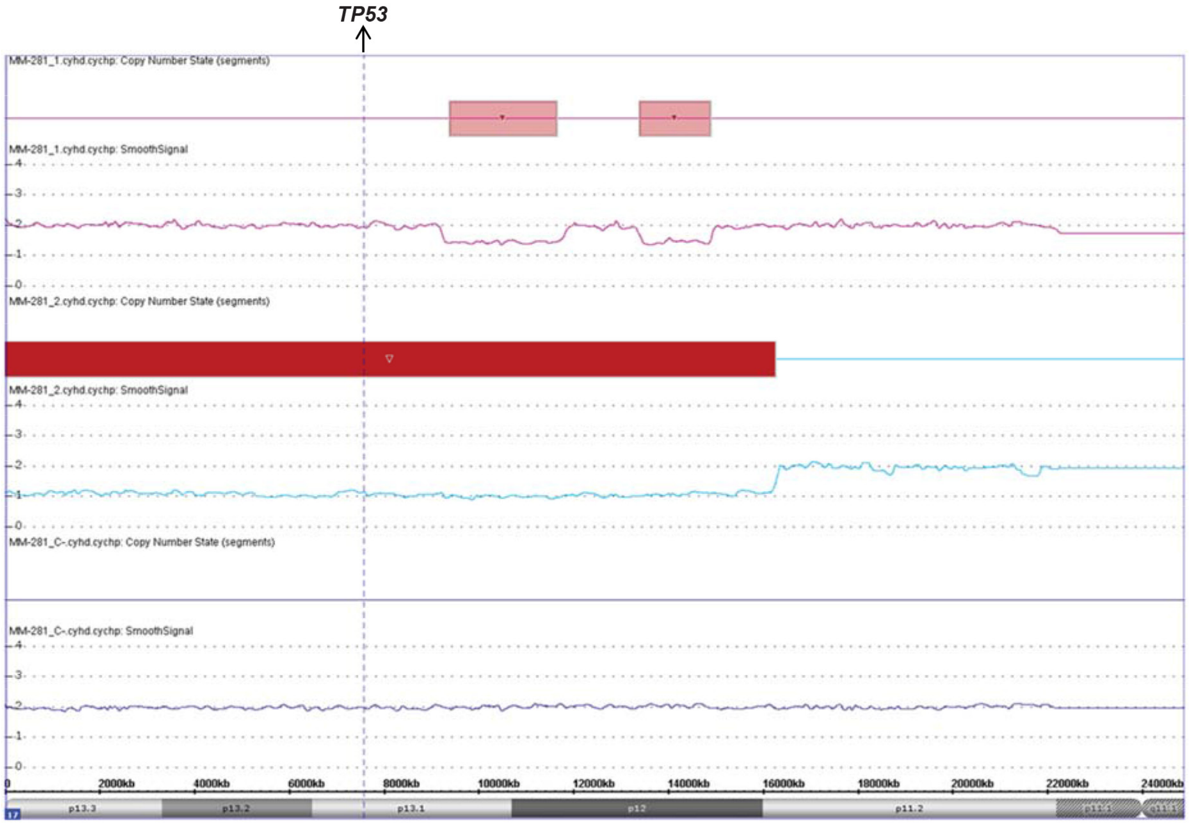

Supplementary Figure S1: Results from analysis with Affymetrix Cytoscan HD array in MM-281. Array-based genotyping was performed on malignant plasma cells collected from the patient at diagnosis (MM-281_1) and at relapse (MM-281_2), and on patient's non-tumor cells (MM-281_C-). Single sample analysis was performed by Chromosome Analysis Suite (Chas) 2.1.0 software. A filter for marker count in defining loss regions was set on 30. Whole-genome DNA profiling detected two interstitial regions of mosaic loss in 17p in the early sample [del(17)(p12p13.1): 2.269 kb, CN state: 1.45; del(17)(p12p12): 1.508 kb, CN state:1.45], and a deletion spanning from 17p11.2 to 17pter of the short arm of the chromosome in the late sample [del(17)(p11.2): 16.260 kb, CN state: 1]. The blue dashed line indicates the *TP53* locus.

Supplementary Table S1: Genome-specific primers for amplicon library preparation

Exon	Primer FWD	Primer REV
4	5′-CCTGGTCCTCTGACTGCTCT-3′	5′-TTCTGGGAAGGGACAGAAGA-3′
4	5′-GTCCAGATGAAGCTCCCAGA-3′	5′-GCCAGGCATTGAAGTCTCAT-3′
5	5′-TCTGTCTCCTTCCTCTTCCTACA-3′	5′-AACCAGCCCTGTCGTCTCT-3′
6	5′-CAGGCCTCTGATTCCTCACT-3′	5′-GCCACTGACAACCACCCTTA-3′
7	5′-CCTGCTTGCCACAGGTCT-3′	5′-GTGATGAGAGGTGGATGGGT-3′
8	5′-GGGAGTAGATGGAGCCTGGT-3′	5′-GCTTCTTGTCCTGCTTGCTT-3′
9	5′-AAAGGGGAGCCTCACCAC-3′	5′-TGTCTTTGAGGCATCACTGC-3′
